# Supplementary material for: High-voltage and dendrite-free zinc-iodine flow battery
Source: Nat Commun. 2024 Jul 24;15:6234. doi: 10.1038/s41467-024-50543-2 (PMC11266666; doi:10.1038/s41467-024-50543-2)
Supplement: Supplementary file 1 — Supplementary Information [file 41467_2024_50543_MOESM1_ESM.pdf]

Supporting Information

High-Voltage and Dendrite-Free Zinc-Iodine Flow Battery

Caixing Wang, Guoyuan Gao, Yaqiong Su, Ju Xie, Dunyong He, Xuemei Wang, Yanrong Wang, Yonggang Wang

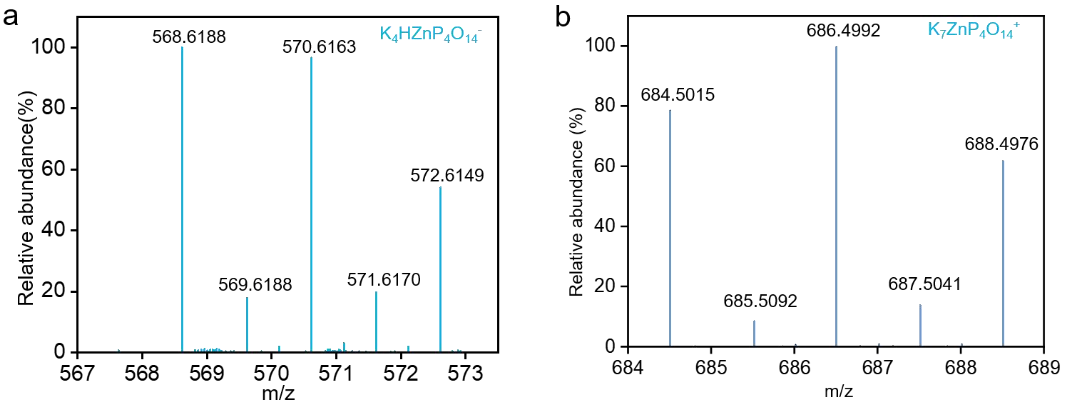

Supplementary Fig. 1 ESI-HRMS of the $Zn(PPi)_2^{6-}$. (a) The peak found at m/z=568.6188 is assigned to $[K_5Zn(P_2O_7)_2]^-$ (calcd:568.6156). (b) The peak found at m/z=684.5015 is assigned to $[K_7Zn(P_2O_7)]^-$ (calcd: 684.4989).

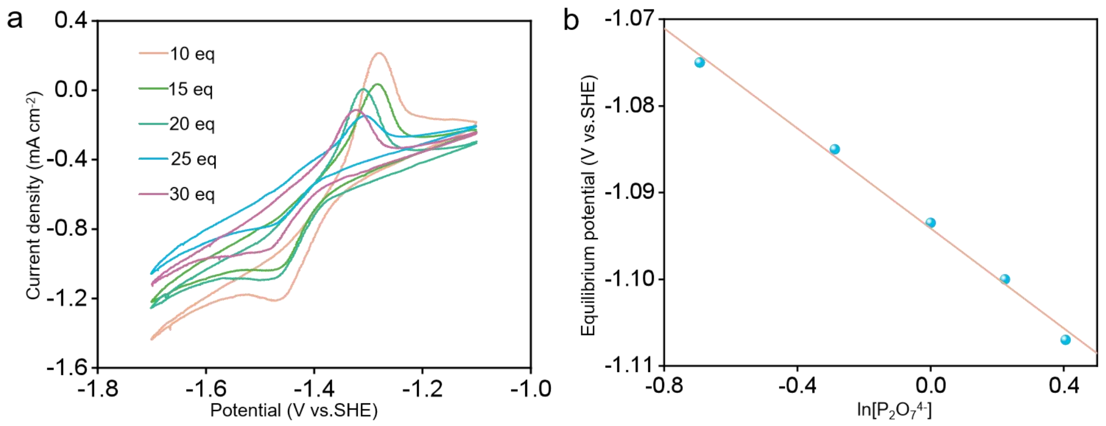

Supplementary Fig. 2 (a) CV curves of the chelated solutions on a Zn foil electrode at a scan rate of 0.1 mV s^{-1}. The concentration of Zn^{2+} is 0.05 mM, and the concentration ratio of $[PPi^{4-}]$:$[Zn^{2+}]$ is ranging from 10:1 to 30:1. (b) The equlibrium potential derived from **a** vs. ln $[PPi^{4-}]$.

For the 0.1 M $Zn(PPi)_2^{6-}$ negolyte (0.1 M $ZnCl_2$ + 0.3 M K_4PPi) used for CV test in **Figure 3a**, the concentration of Zn^{2+} at the equilibrium sate was set as "*x*", the concentration of $Zn(PPi)_2^{6-}$ was 0.1-*x*, and the concentration of PPi^{4-} was 0.1+2*x*.

	Zn^{2+} +	$2PPi^{4-}$ ↔	$Zn(PPi)_2^{6-}$
Initial state	0.1	0.3	0
Equilibrium sate	*x*	0.1+2*x*	0.1-*x*

According to the stability constant of $Zn(PPi)_2^{6-}$ (Log K = 11.0), the equation is given as follows:

$$\frac{0.1 - x}{x(0.1 + 2x)^2} = 10^{11}$$

The *x*-value is estimated to be 1×10^{-10}. It means that the concentration of Zn^{2+} in 0.1 M $Zn(PPi)_2^{6-}$ negolyte is 1×10^{-10} M. Considering the Nernst Equation:

$$\varphi_{Zn(PPi)_2{}^{6-}/Zn} = \varphi_{Zn^{2+}/Zn} + \frac{RT}{nF}\ln[Zn^{2+}] = -0.76\ V + 0.029\log[Zn^{2+}]$$

It is believed such a low concentration (1×10^{-10} M) of Zn^{2+} well explains the negative shift of the Zn plating/stripping potential in the CV tests.

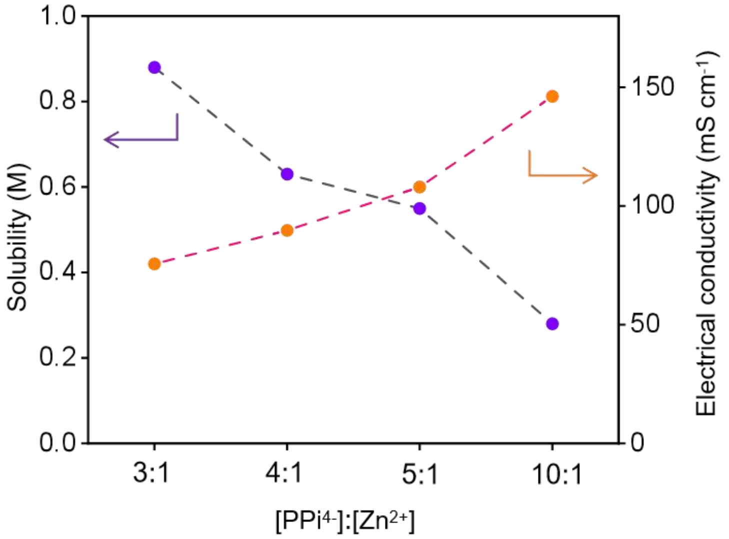

Supplementary Fig. 3 The solubility and conductivity of $Zn(PPi)_2^{6-}$ at different concentration ratio of PPi^{4-} and Zn^{2+}.

Solubility test: The $Zn(PPi)_2^{6-}$ solution with a $[PPi^{4-}]$:$[Zn^{2+}]$ molar ratio of 3:1 was prepared with 40 mL of 1 M $ZnBr_2$ and 25 mL of 3 M K_4PPi, and then concentrated to 45 mL at 50 °C under reduced pressure. The solubility is thus calculated to be $\frac{1M\times 40\ mL}{45\ mL} = 0.9\ M$. The other solubilities of $Zn(PPi)_2^{6-}$ solutions with different $[PPi^{4-}]$:$[Zn^{2+}]$ molar ratios can be calculated by the same method.

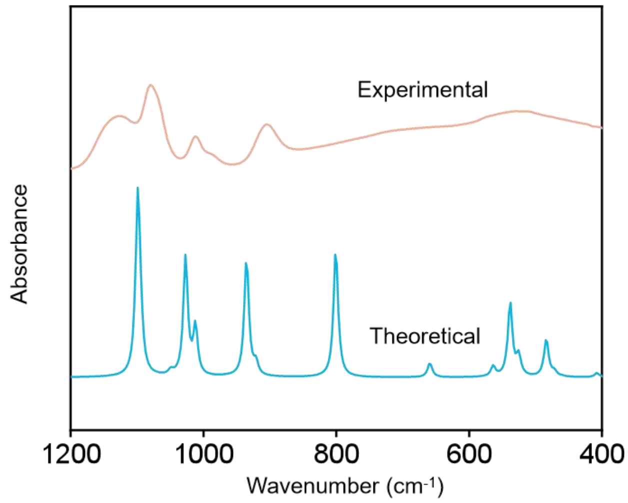

Supplementary Fig. 4 The experimental and theoretical FTIR spectra of $Zn(PPi)_2^{6-}$ ion in the region from 1200 to 400 cm^{-1}, respectively.

a

b

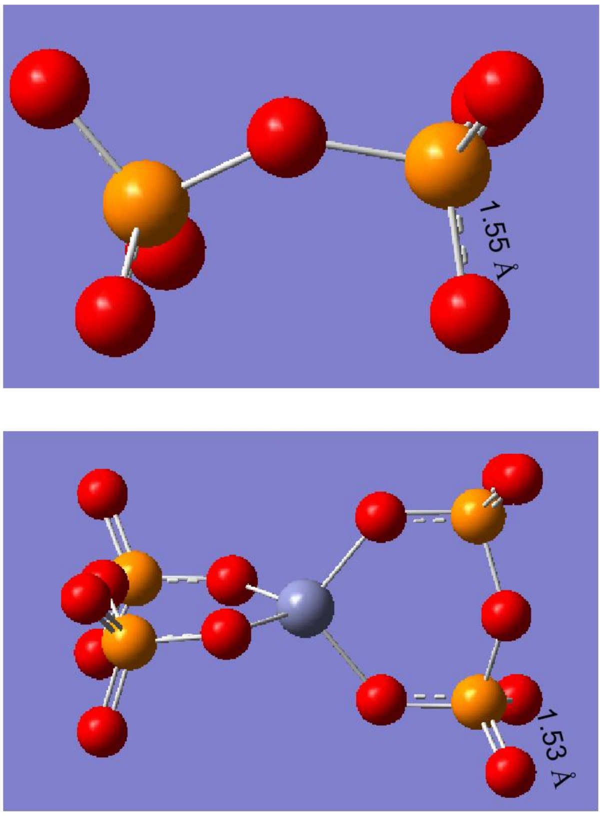

Supplementary Fig. 5 (a) The optimized bond length of P=O for PPi^{4-}. (b) The optimized bond length of P=O for $Zn(PPi)_2^{6-}$.

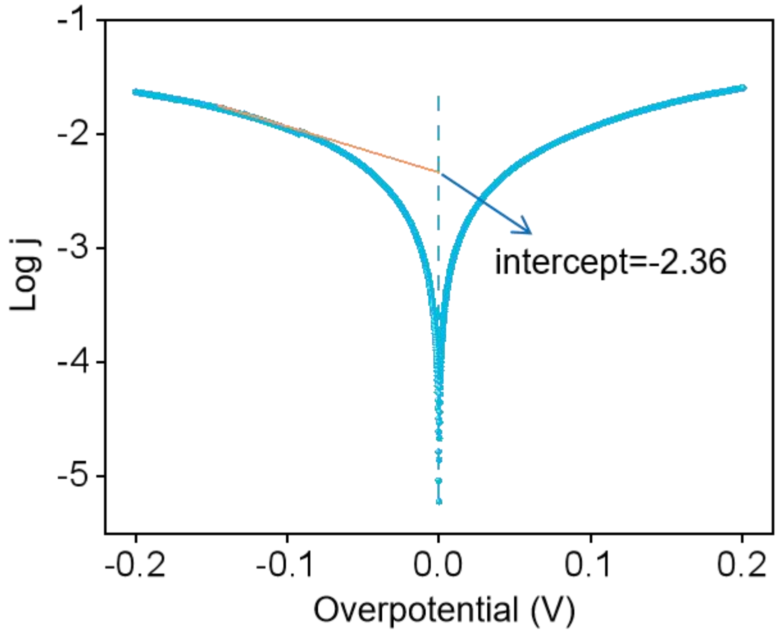

Supplementary Fig. 6 Tafel plots for Zn plating/stripping in 0.2 M $ZnBr_2$ solution at a scan rate of 0.1 mV s^{-1}.

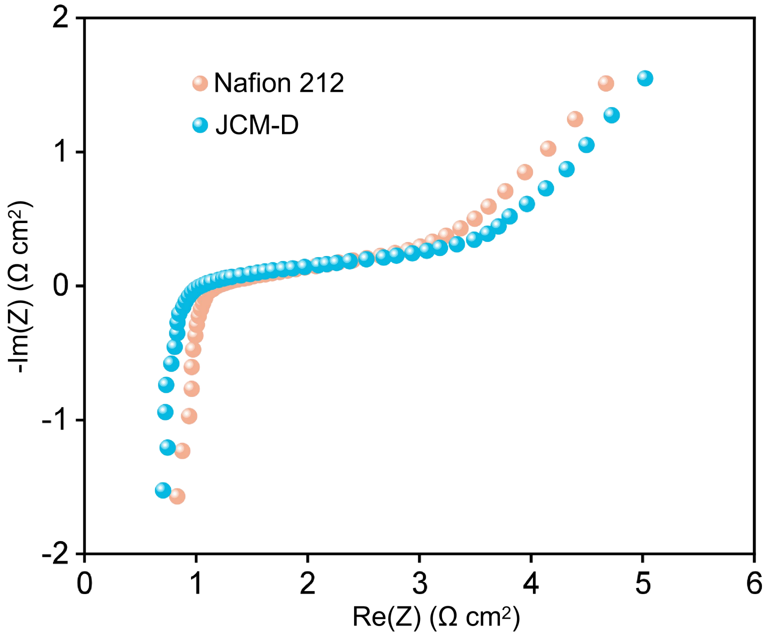

Supplementary Fig. 7 The area resistance of JCM-D membrane and Nafion 212 membrane in 1 M KCl solution, respectively.

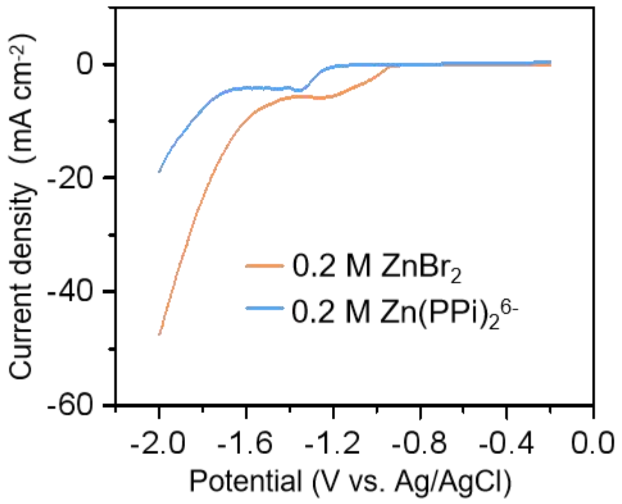

Supplementary Fig. 8 LSV profiles of the 0.2 M $ZnBr_2$ and 0.2 M $Zn(PPi)_2^{6-}$ negolytes on carbon paper (1 cm^{-2}) at a scan rate of 5 mV s^{-1}.

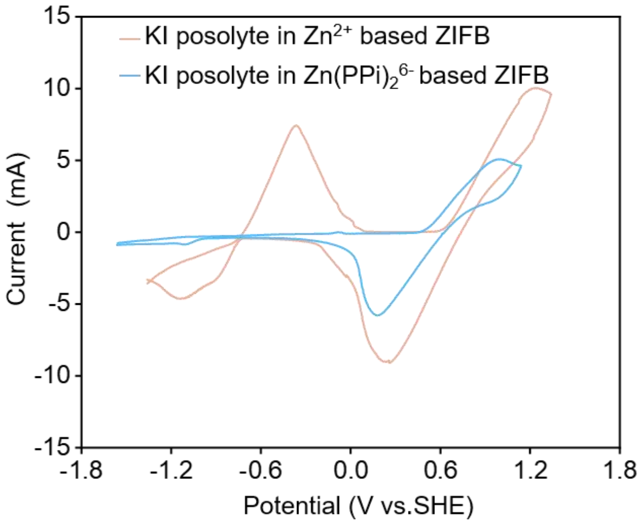

Supplementary Fig. 9 CV curves of KI posolytes for two ZIFBs at a scan rate of 100 mV s^{-1}.

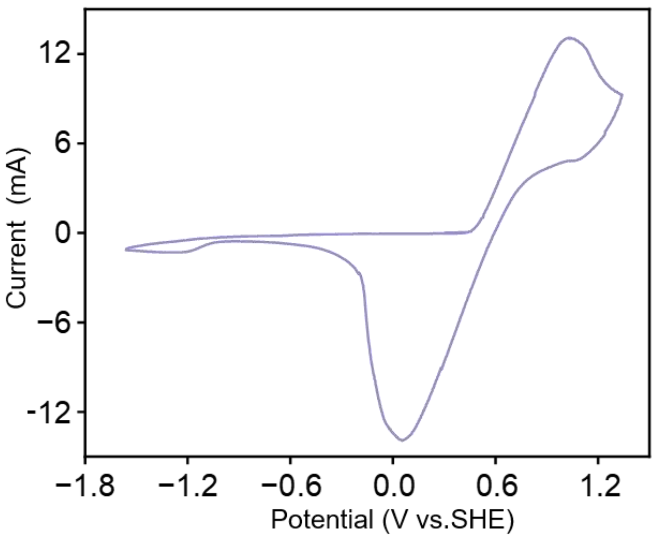

Supplementary Fig. 10 CV curve of 1.0 M KI in 1 M KCl solution at a scan rate of 100 mV s^{-1}.

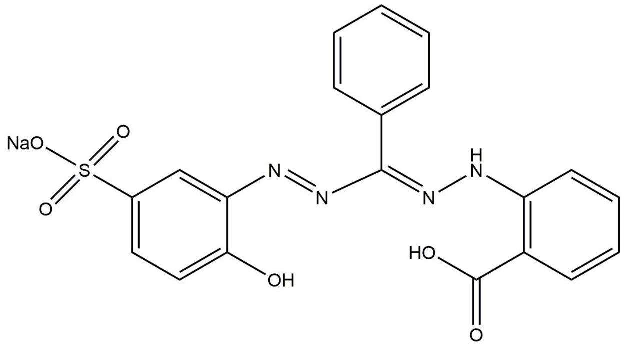

Supplementary Fig. 11 The structure of zincon monosodium salt ($C_{20}H_{15}N_4NaO_6S$), which is served as a complex reagent for the detection of Zn^{2+}.

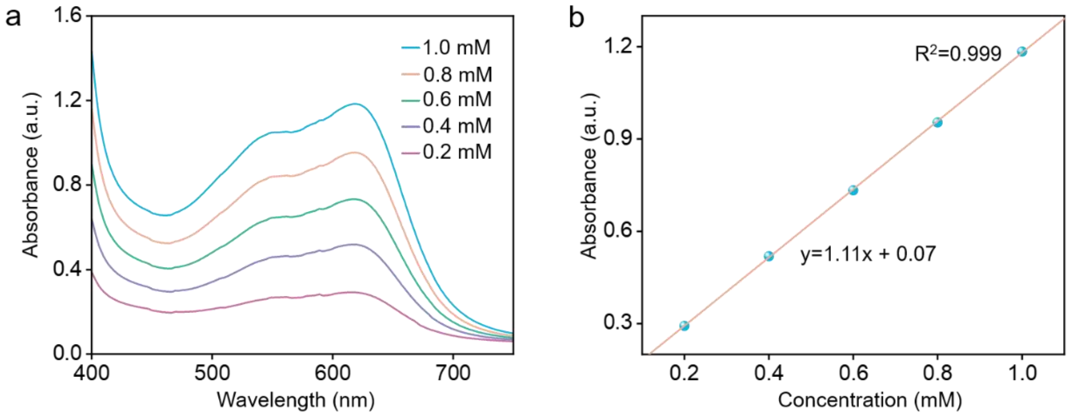

Supplementary Fig. 12 (a) UV-vis spectra of $ZnBr_2$ solution with zincon monosodium salt, and the concentration of Zn^{2+} vary from 0.2 mM to 1.0 mM, respectively. (b) Absorbance *vs.* concentration fitting plots of (a) at λ_{620} nm.

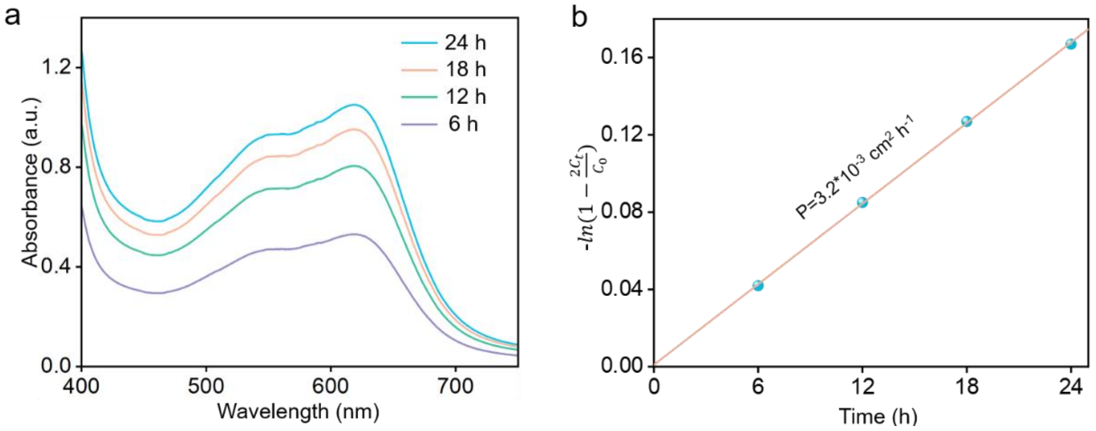

Supplementary Fig. 13 (a) UV-vis spectra of $ZnBr_2$ solution permeated through a JCM-D cation membrane, saturated zincon monosodium salt is added in the reference cell. (b) The permeability of Zn^{2+} ions.

The saturated zincon monosodium salt reagent is added to the right-hand cell in advance, and if trace amounts of zinc ions diffuse from the left compartment, the zincon monosodium salt (yellow in colour) will coordinate with the Zn^{2+} and the solution will appear in red colour.

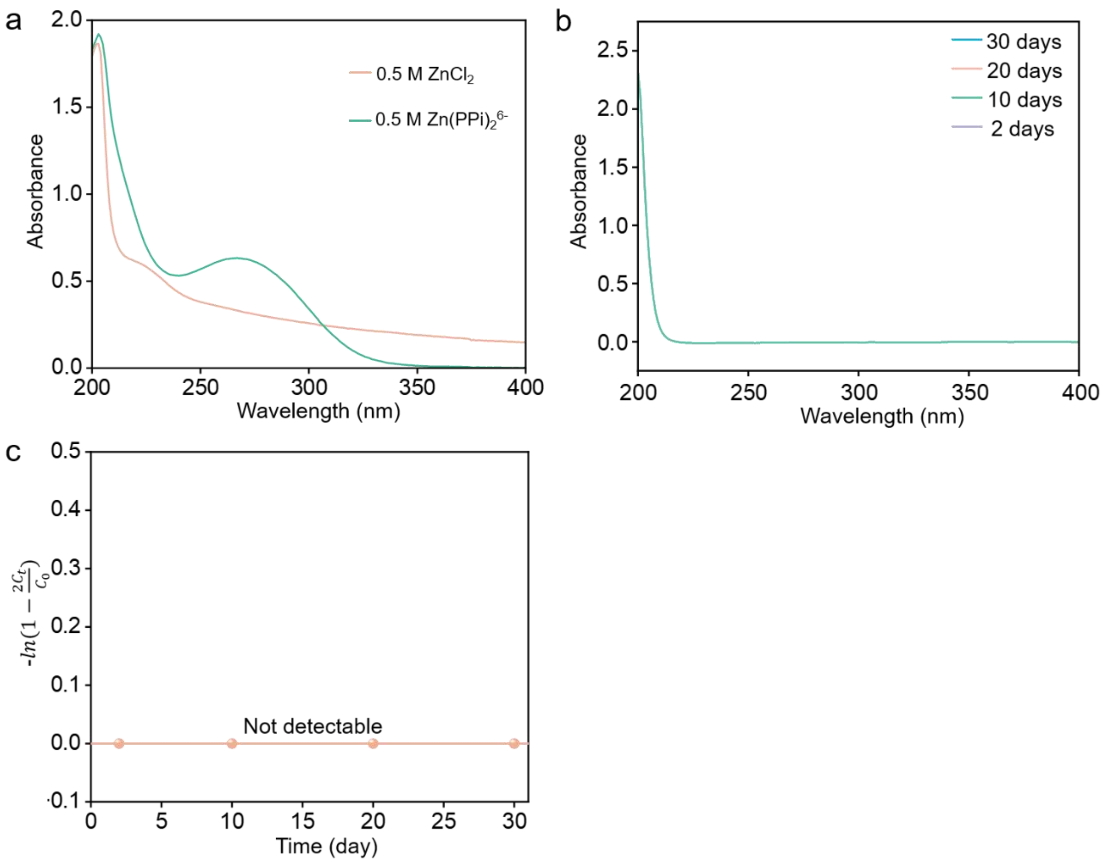

Supplementary Fig. 14 (a) UV-vis spectra of $ZnCl_2$ solution and $Zn(PPi)_2^{6-}$ solution, respectively. (b) UV-vis spectra of $Zn(PPi)_2^{6-}$ solution permeated through a JCM-D cation membrane. (c) The permeability of $Zn(PPi)_2^{6-}$ ions.

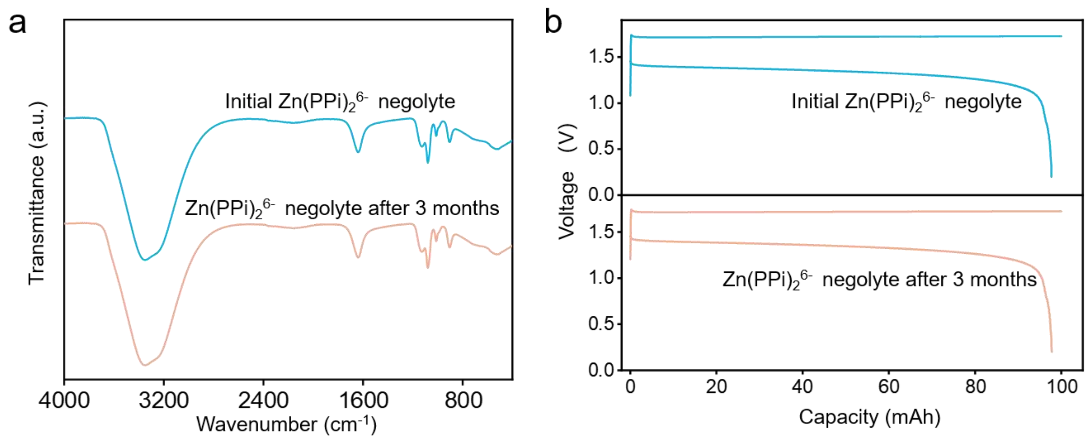

Supplementary Fig. 15 (a) The ATR-FITR spectra of as-prepared $Zn(PPi)_2^{6-}$ negolyte in initial state and after placed in air for three months. (b) The initial GCD curves of ZIFBs based on as-prepared $Zn(PPi)_2^{6-}$ negolyte and $Zn(PPi)_2^{6-}$ negolyte placed in air for three months, respectively.

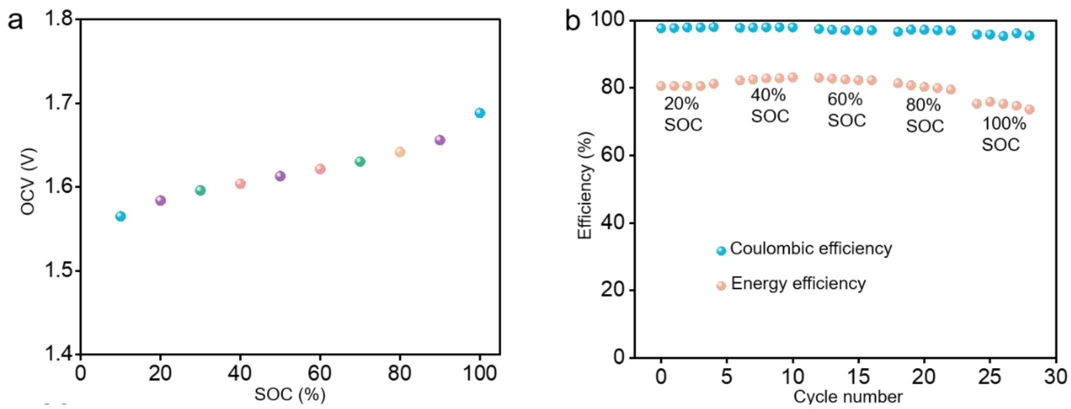

Supplementary Fig. 16 Cell performance of 0.2 M $Zn(PPi)_2^{6-}$ based ZIFBs. (a) The open-circuit voltage (OCV) of ZIFBs at different SOCs. (b) Efficiency of the cell at different SOCs.

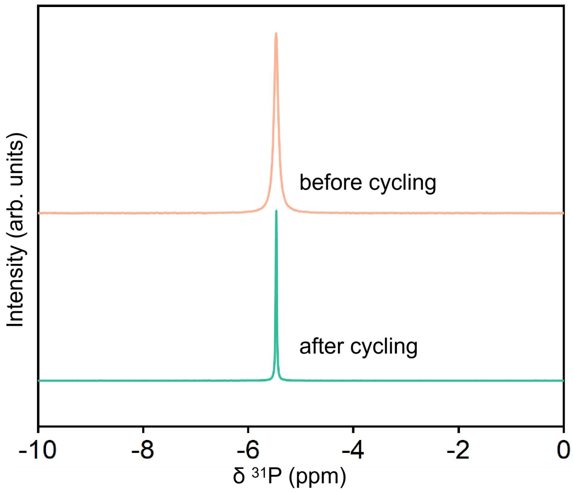

Supplementary Fig. 17 ^{31}P NMR spectra of 0.8 M $Zn(PPi)_2^{6-}$ negolyte before and after cycling, respectively.

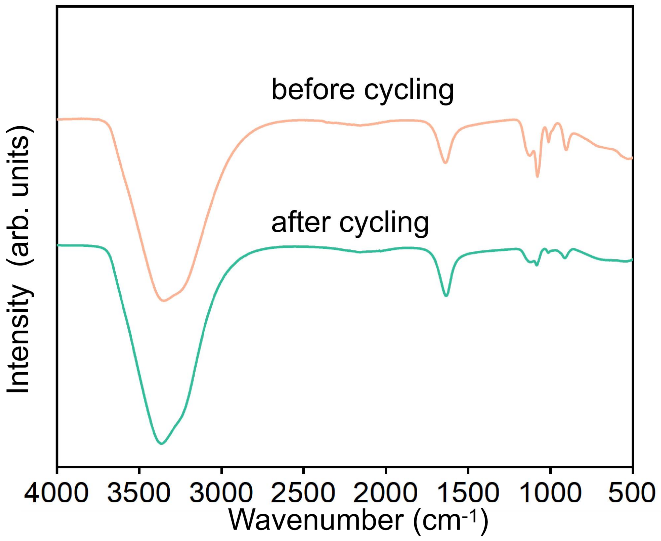

Supplementary Fig. 18 FT-IR spectra of 0.8 M $Zn(PPi)_2^{6-}$ negolyte before and after cycling, respectively.

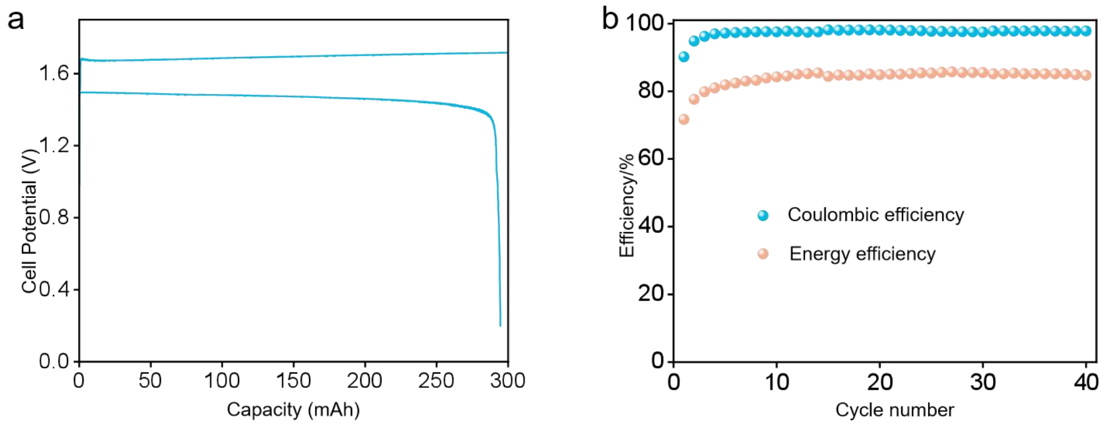

Supplementary Fig. 19 (a) The GCD profile of 0.8 M $Zn(PPi)_2^{6-}$ based ZIFB at 80 mA cm^{-2} with deposited Zn areal capacity of 60 mAh cm^{-2} at 10th cycle. (b) Cycle performance of the corresponding ZIFB based on 0.8 M $Zn(PPi)_2^{6-}$ negolyte.

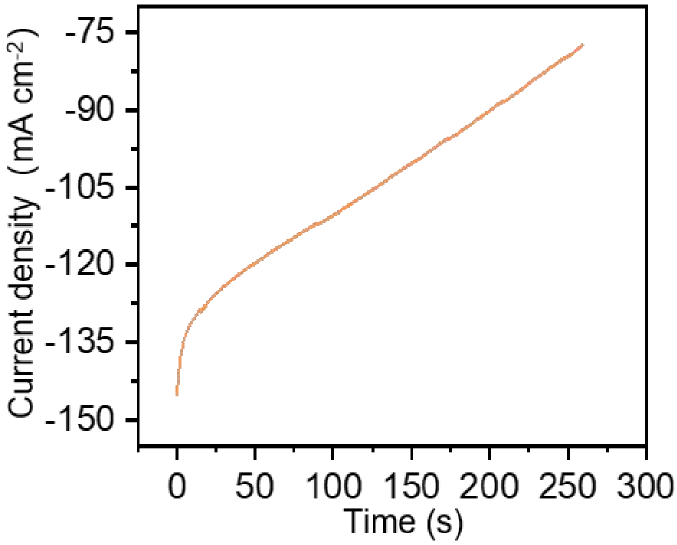

Supplementary Fig. 20 Zinc plating profile in 0.8 M $Zn(PPi)_2^{6-}$ electrolyte using the constant potential polarization method. Carbon felt (1 cm^2) was served as the working electrode, zinc foil (0.2 mm thick) as the counter electrode and Ag/AgCl as the reference electrode, respectively. The plating potential was set at -1.5 V vs. Ag/AgCl.

The number of moles of plated Zn per second at 200 mA cm^{-2} on the electrode (5 cm^2) is:

$$\frac{0.2\ \mathrm{A\ cm^{-2}} \times 5\ \mathrm{cm^2} \times 1\ \mathrm{s}}{2\times\ 96485\ C\ mol^{-1}} = \frac{1\ C}{2 \times 96485\ C\ mol^{-1}} = 0.52 \times 10^{-6}\ \mathrm{mol}$$

The volume of negolyte in the cell chamber is 0.2 cm $\times$ 5 cm^2 = 1 mL. The total amount of $Zn(PPi)_2^{6-}$ ions is 0.8 M × 1 mL = 0.8×10^{-3} mol. It means that the plating current density of 200 mA cm^{-2} could be achieved as long as 0.65% of the $Zn(PPi)_2^{6-}$ electrolyte in the battery chamber dissociates to Zn^{2+} per second.

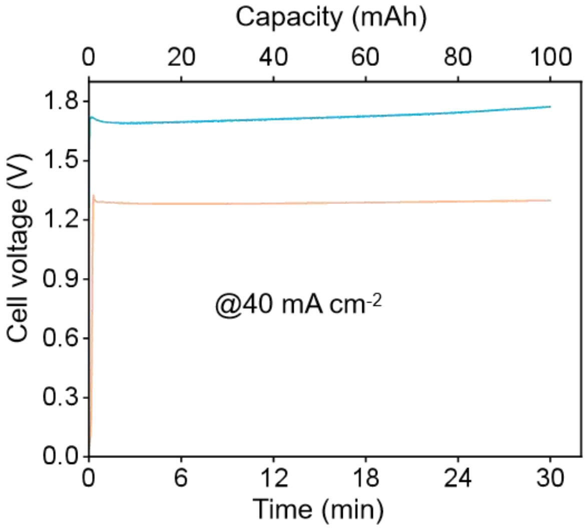

Supplementary Fig. 21 Charge profile of two neutral ZIFBs at 40 mA cm^{-2} using 0.2 M $ZnBr_2$ (light blue) and 0.2 M $K_6Zn(PPi)_2$ (orange) negolytes with a deposited Zn areal capacity of 20 mAh cm^{-2}, respectively.

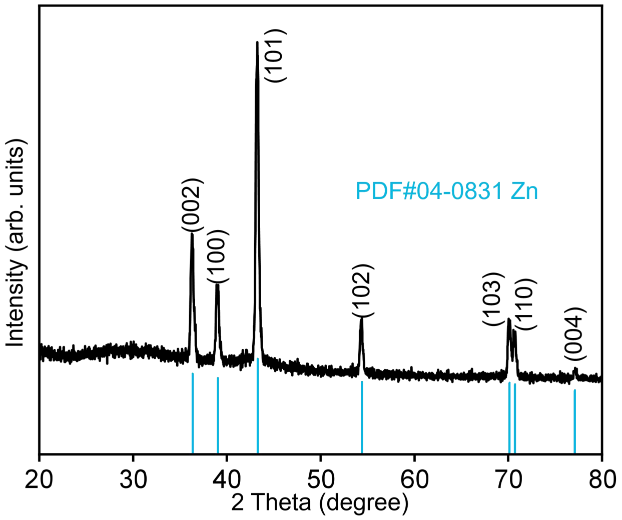

Supplementary Fig. 22 XRD pattern of deposited Zn (areal capacity of 20 mAh cm^{-2}) on carbon felt using 0.2 M $ZnBr_2$ negolyte.

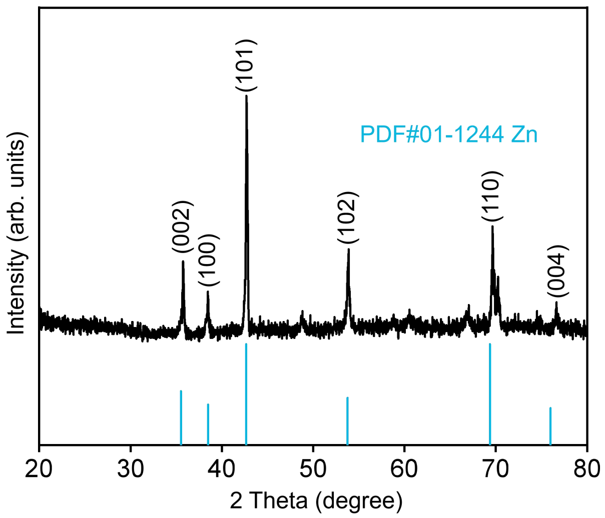

Supplementary Fig. 23 XRD pattern of deposited Zn (areal capacity of 20 mAh cm^{-2}) on carbon felt using 0.2 M $Zn(PPi)_2^{6-}$ negolyte.

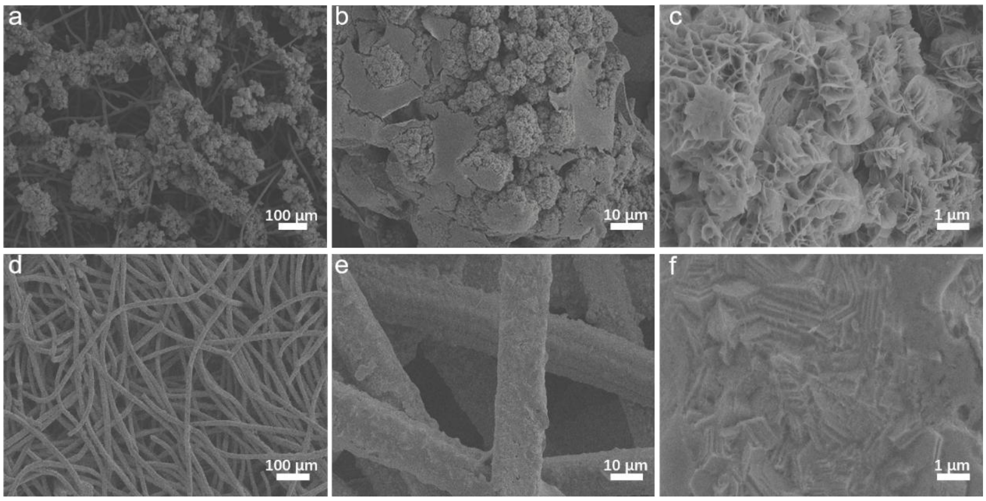

Supplementary Fig. 24 SEM images of deposited Zn (20 mAh cm^{-2}) on the surface of carbon felts using 0.2 M $ZnBr_2$ negolyte (a-c) and $Zn(PPi)_2^{6-}$ negolyte (d-f), respectively.

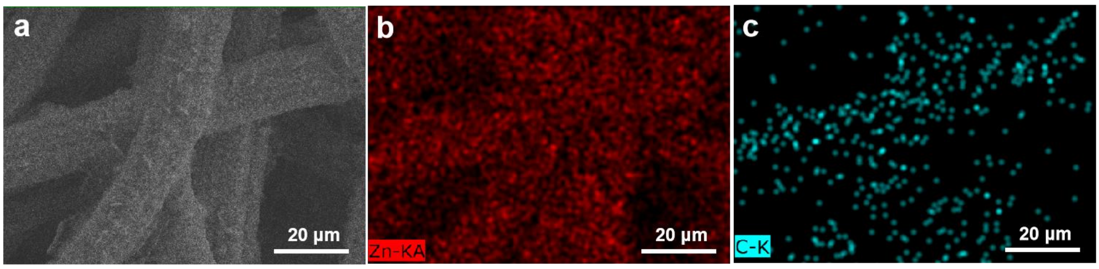

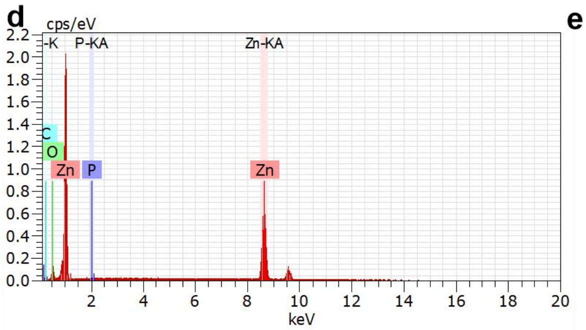

e

Element	wt.%	At.%
Zn	92.22	68.9
P	0.22	0.35
C	7.56	30.76

Supplementary Fig. 25 SEM image (a) and corresponding elemental mapping (b,c) and EDX analyses (d,e) of deposited Zn (areal capacity of 20 mAh cm^{-2}) on carbon felt using 0.2 M $Zn(PPi)_2^{6-}$ negolyte.

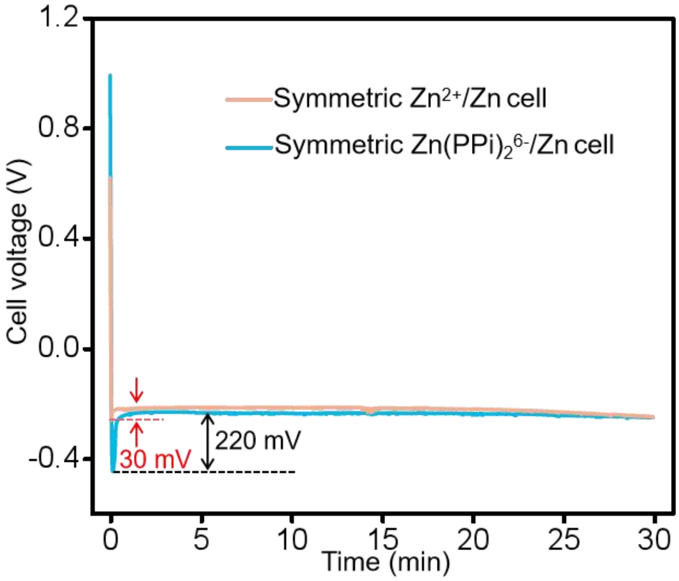

Supplementary Fig. 26 Symmetric zinc-based RFBs based on 0.2 M $ZnBr_2$ (light blue line) and 0.2 M $K_6Zn(PPi)_2$ (orange line) negolytes operated at 40 mA cm^{-2} using noncharged filter membrane. The capacity of pre-deposited Zn on the carbon felt at the positive side is 200 mAh.

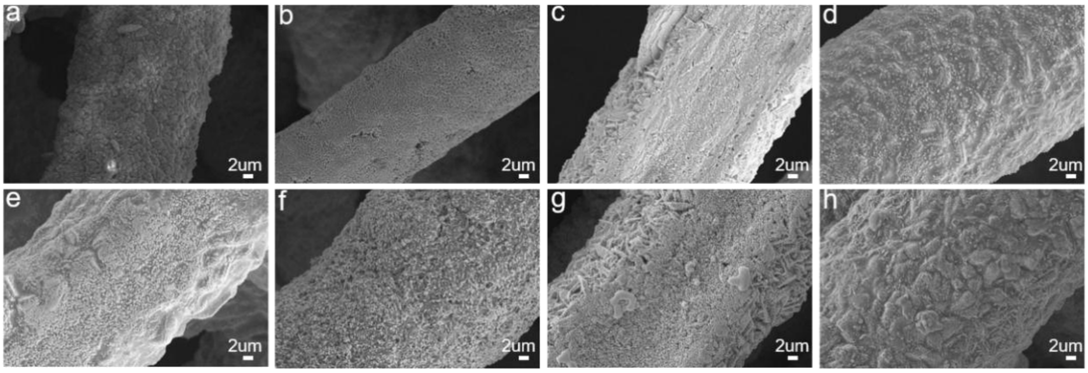

Supplementary Fig. 27 The SEM images of deposited Zn on the surface of carbon felt fiber with areal capacities range from 40 mAh cm^{-2} to 180 mAh cm^{-2} in 0.8 M $Zn(PPi)_2^{6-}$ negolyte at a current density of 80 mA cm^{-2}.

In conventional aqueous electrolytes containing large amounts of free Zn^{2+}, the formation of hydrated Zn^{2+} is inevitable. During the Zn plating process, H_2O molecules in the solvated structure of Zn^{2+} can gain electrons, resulting in HER. Such undesired HER not only reduces the Coulombic efficiency, but also leads to inhomogeneous Zn plating. In the subsequent Zn plating, the top effect of these inhomogeneous Zn deposits can aggravate the dendrite growth. In the $Zn(PPi)_2^{6-}$ electrolyte (e.g., 0.1 M $ZnCl_2$ + 0.3 M K_4PPi), the concentration of free Zn^{2+} is very low (~10^{-10} M), and therefore H_2O molecules remain in the free solvent network or in conjunction with K^+ (1.2 M),[1] rather than generating hydrated Zn^{2+}. As a result, the $Zn(PPi)_2^{6-}$ electrolyte mitigates the undesired HER and promotes smooth Zn plating.

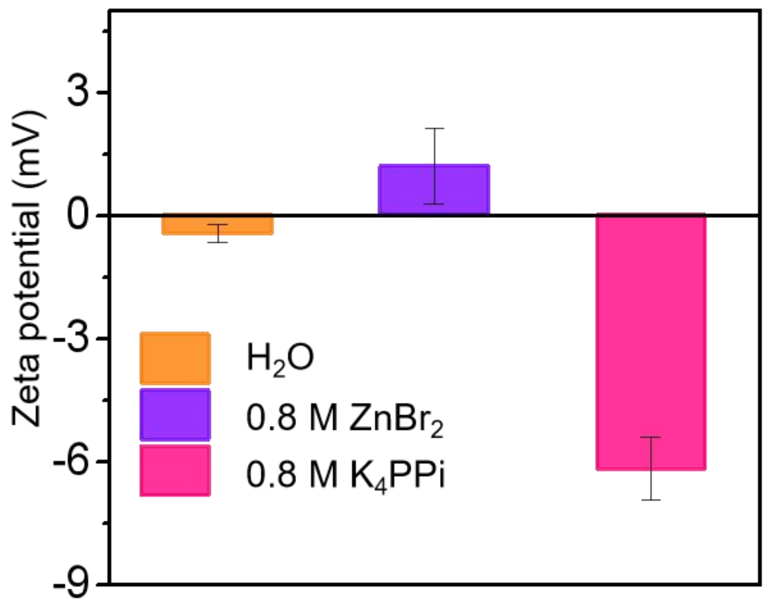

Supplementary Fig. 28 Zeta potential of zinc powder in various solutions.

Supplementary Table 1. Comparison of electrochemical performance for different ZIFBs.

Posolyte	Negolyte	Membrane	Cell voltage (V)	Current density (mA cm^{-2})	Deposited Zn areal capacity (mAh cm^{-2})	Cycle number	Ref.
3 M ZnI_2	3 M ZnI_2	-	1.30	5	-	30	2
3.5 M ZnI_2	3.5 M ZnI_2	Nafion 115 (127 um)	1.30	10	42	40	3
3.5 M ZnI_2 + 1.75 M $ZnBr_2$	3.5 M ZnI_2 + 1.75 M $ZnBr_2$	Nafion 117 (183 um)	1.35	10	35	50	4
6.5 M NH_4I + 3.25 M NH_4Cl	$ZnCl_2$ + NH_4Cl (with Zn plate)	Nafion 117	1.30	10	-	2500	5
1.5 M ZnI_2 + 1.5 M KI	1.5 M ZnI_2 + 1.5 M KI (with Zn plate)	Nafion 115	1.30	10	-	10	6
6 M KI-6 M I_2	6 M KOH (with Zn plate)	Nafion 117	1.80	10	-	70	7
1 M KI + 5.3%PVP	0.5 M $ZnCl_2$ + 3 M KCl	PSA	1.38	20	3.6	600	8
5 M ZnI_2 + 2.5 M NH_4Br	5 M ZnI_2 + 2.5 M NH_4Br	Nafion 115	1.30	40	7.8	100	9
6 M KI + 3 M $ZnBr_2$ +2 M KCl	6 M KI + 3 M $ZnBr_2$ + 5 wt% PEG300	Nafion 115	1.30	40	10	200	10
1 M KI + 0.5 M $ZnBr_2$ + 2 M KCl	1 M KI + 0.5 M $ZnBr_2$ + 2 M KCl	C7S3 (30 um)	1.30	60	-	200	11
6 M KI + 3 M $ZnBr_2$ +2 M KCl	6 M KI + 3 M $ZnBr_2$ +2 M KCl	C@porous polyolefin	1.33	80	55	1000	12
6 M KI + 3M $ZnBr_2$	6 M KI + 3 M $ZnBr_2$	Nafion@porous polyolefin	1.35	80	23	500	13
6 M KI + 3 M $ZnBr_2$	6 M KI + 3 M $ZnBr_2$	Nafion 115	1.30	100	25	120	14
-	1 M ZnI_2 + 7.5% ACN	Nafion 115	1.30	100	-	170	15
4 M KI + 2 M KCl	0.8 M $K_6Zn(PPi)_2$ + 1.6 M KCl	JCM-D (30 um)	1.61	200	40	250	This work

Supplementary References

1. Yang, C. et al. All-temperature zinc batteries with high-entropy aqueous electrolyte. *Nat. Sustain.* **6**, 325-335 (2023).
2. Aubin, C. A. et al. Electrolytic vascular systems for energy-dense robots. *Nature* **571**, 51-57 (2019).
3. Li, B. et al. Ambipolar zinc-polyiodide electrolyte for a high-energy density aqueous redox flow battery. *Nat. Commun.* **6**, 6303 (2015).
4. Weng, G.-M., Li, Z., Cong, G., Zhou, Y., Lu, Y.-C. Unlocking the capacity of iodide for high-energy-density zinc/polyiodide and lithium/polyiodide redox flow batteries. *Energy Environ. Sci.* **10**, 735-741 (2017).
5. Mousavi, M. et al. Decoupled low-cost ammonium-based electrolyte design for highly stable zinc–iodine redox flow batteries. *Energy Storage Mater.* **32**, 465-476 (2020).
6. Chakraborty, M., Murcia-López, S., Morante, J. R., Andreu, T. Structural influence of the anode materials towards efficient Zn deposition/dissolution in aqueous zn-iodide flow batteries. *J. Electrochem. Soc.* **168**, 040532 (2021).
7. Zhang, J. et al. An all-aqueous redox flow battery with unprecedented energy density. *Energy Environ. Sci.* **11**, 2010-2015 (2018).
8. Yang, J., Song, Y., Liu, Q., Tang, A. High-capacity zinc–iodine flow batteries enabled by a polymer–polyiodide complex cathode. *J. Mater. Chem. A* **9**, 16093-16098 (2021).
9. Jian, Q. P., Wu, M. C., Jiang, H. R., Lin, Y. K., Zhao, T. S. A trifunctional electrolyte for high-performance zinc-iodine flow batteries. *J. Power Sources* **484**, 229238 (2021).
10. Jin, S. et al. Stabilizing zinc electrodeposition in a battery anode by controlling crystal growth. *Small* **17**, 2101798 (2021).
11. Gao, L., Ding, Y., He, G., Yu, G. Bio-derived and cost-effective membranes with high selectivity for redox flow batteries based on host–guest chemistry. *Small* **18**, 2107055 (2022).
12. Xie, C., Zhang, H., Xu, W., Wang, W., Li, X. A long cycle life, self-healing zinc-iodine flow battery with high power density. *Angew. Chem. Int. Ed.* **57**, 11171-11176 (2018).
13. Xie, C., Liu, Y., Lu, W., Zhang, H., Li, X. Highly stable zinc–iodine single flow batteries with super high energy density for stationary energy storage. *Energy Environ. Sci.* **12**, 1834-1839 (2019).
14. Jin, S. et al. Designing interphases for practical aqueous zinc flow batteries with high power density and high areal capacity. *Sci. Adv.* **8**, eabq4456 (2022).
15. Zhao, Y. et al. Accelerating the dissolution kinetics of iodine with a cosolvent for a high-current zinc–iodine flow battery. *J. Mater. Chem. A* **10**, 14090-14097 (2022).
